# Advancing environmentally sustainable learning health systems: Perspectives from a Canadian health center

**DOI:** 10.1002/lrh2.10470

**Published:** 2025-01-28

**Authors:** Brittany V. Barber, Douglas Sinclair, Christine Cassidy

**Affiliations:** ^1^ School of Nursing Dalhousie University Halifax Nova Scotia Canada; ^2^ IWK Health Centre Halifax Nova Scotia Canada

**Keywords:** environmental scan, health system change, learning health systems, sustainable health care

## Abstract

**Background:**

There is increasing demand for health systems to reduce greenhouse gas emissions and invest in climate‐resilient health care. Coordinating organizational structures and processes for reducing health system emissions presents challenges. Learning health systems, defined as systems that seek to continuously generate and apply evidence, innovation, quality, and value in health care, can guide health systems with planning organizational structures and processes to advance environmentally sustainable healthcare. The purpose of this research is to gather in‐depth insight from key health system leaders and healthcare professionals to identify challenges and recommendations for planning environmentally sustainable learning health systems.

**Methods:**

Environmental scan methods were used, comprising jurisdictional literature review and informal discussions with key informants at one tertiary care center in Nova Scotia, Canada. Key informants were asked to describe challenges of coordinating environmentally sustainable health system structures and processes, and recommendations to advance planning for environmentally sustainable learning health systems. Deductive thematic analysis was used to categorize challenges and recommendations into seven characteristics of a learning health system framework.

**Results:**

Informal discussions with 16 key informants provide detailed descriptions of 7 challenges and recommendations for planning and coordinating organizational structures and processes to advance environmentally sustainable learning health systems. Health system challenges include limited patient and community engagement, no systematic approach to measuring and monitoring emissions data, and limited knowledge of sustainability co‐benefits and strategies for mobilizing sustainable organizational change. Recommendations include engaging patients and communities in co‐creation of sustainable healthcare, monitoring of emissions data identifying high‐impact areas for action, and well‐coordinated leadership supporting sustainable policies, procedures, and decision‐making in practice.

**Conclusion:**

Learning health systems provide structure for establishing critical processes to adapt to routinely collected data through rapid cycle improvements, and operationalization of value‐based health care that prioritizes health outcomes, reduction of costs, and mitigating environmental impacts.

## 1 INTRODUCTION

High‐performing and environmentally sustainable health systems are critical for meeting future challenges and health care demands for populations living longer with higher complex health needs^1^ and increasing risk of climate‐related health conditions (i.e., heat stress, respiratory, and cardiovascular disease from reduced air quality).^2^ Climate change poses significant public health and health system challenges, such as increased demand for health services due to chronic and acute health impacts from vector‐borne diseases, heat‐related illness, and injury from severe weather.^3^, ^4^ Future generations are at highest risk for climate‐related health conditions,^2^ an estimated 1 in 5 new cases of childhood asthma in Canada is caused by traffic‐related pollution.^5^

As climate change worsens, so do its effects on health systems, such as the increasing severity of weather extremes causing damage to health care infrastructure and interference with supply chains.^6^ Ironically, health systems globally are significant contributors to climate change, generating an estimated 5% of global emissions, with greenhouse gas emissions (GHG) equivalent to 514 coal‐fired power plants annually.^7^ Carbon dioxide is the primary GHG emitted, accounting for approximately 86% of GHG emissions, hence why carbon emissions and carbon footprint are often used interchangeably when discussing GHG emissions.^8^, ^9^ Health system GHG emissions are produced from direct and indirect sources.^10^ Direct sources account for between 10% and 30% of emissions generated by medical gases and local transport freight. Indirect sources account for the largest share, between 50% and 70% of emissions generated by purchased or acquired electricity, steam, heat, and cooling, health care supply chain, waste, and pharmaceuticals.^9^ Health systems in the United States, China, and the European Union produce the most significant GHG emissions globally.^7^ However, per capita, Australia, Canada, Switzerland, and the United States are among the top four worst health system GHG emitters globally, producing between 30 and 57 times more emissions per person than India.^7^

In the last decade, research has increasingly focused on using Life Cycle Assessment methods^11^ to quantify environmental impacts and GHG emissions from supply chain emissions, such as single‐use versus reusable medical supplies.^12^ Assessing GHG emissions at the point of care is critical for building the evidence base of clinical interventions and practice changes that could bring about a significant reduction in GHG emissions. However, targeting micro‐level health care interventions makes it challenging to advance large‐scale health system change. To address complex, organizational‐ and system‐level environmental sustainability challenges, a systems‐thinking approach is required to coordinate action and mobilize climate‐resilient organizational change.^13^, ^14^

Strong governance structures for achieving net‐zero health systems are demonstrated by the United Kingdom's National Health Service's centralized approach and Germany's Statutory Health Insurance's decentralized approach.^13^ Despite leading governance approaches for decarbonizing health care, it is unclear *how* organizations coordinate a strategy to implement net‐zero health care. The World Health Organization's operational framework^15^ defines six building blocks of climate‐resilient and low‐carbon health systems including: (1) leadership and governance, (2) health workforce, (3) health information systems, (4) essential medical products and technologies, (5) service delivery, and (6) financing. This operational framework outlines recommendations for health systems to respond to climate‐related public health threats including managing health system risks related to climate change, improving health system capacity to respond to climate‐related health risks, and developing long‐term organizational strategies to continually monitor and adapt health system performance in relation to climate‐related risks.^15^ These system‐level recommendations are generalizable to health systems globally; however, health organizations are required to develop their own tailored approach to enhance successful uptake of climate‐resilient and low‐carbon health care. Without a structured approach guiding organizational change, health centers are challenged with establishing structures and processes for generating and applying evidence, requiring every health center to re‐create an organizational strategy and implementation plan without any prior progress or advantage. A learning health systems (LHS) framework can help address this gap, providing a structured approach to guide organizations with leveraging evidence to respond to environmental sustainability challenges through cycles of continuous improvement.^16^

LHS, defined as systems that seek to continuously generate and apply evidence, innovation, quality, and value in health care,^17^ offers a solution to transform high‐performing, climate‐resilient and low‐carbon health care. A LHS leverages routinely collected data on care delivery, as it responds to changing evidence by incorporating lessons learned through rapid cycle improvements on a continuous basis.^16^ A widely accepted definition of LHS from the Institute of Medicine describes a system whereby “science, informatics, incentives, and culture are aligned for continuous improvement and innovation, with best practices seamlessly embedded in the delivery process, patients and families active participants in all elements, and new knowledge captured as an integral by‐product of the delivery experience.”^17^ Many LHS frameworks have been proposed,^18^ depicting key characteristics and elements of LHS functioning including common values, governance, culture, data exchange, and ongoing cycles of learning. A Canadian LHS framework^19^ defines seven characteristics including: (1) engaged patients; (2) digital capture, linkage and timely sharing of relevant data; (3) timely production of research evidence; (4) appropriate decision supports; (5) aligned governance, financial and delivery arrangements; (6) culture of rapid learning and improvement; and (7) competencies for rapid learning and improvement. Furthermore, a value‐creating LHS framework proposed by Menear et al.^20^ defines four key elements: (1) core values; (2) pillars and accelerators; (3) processes; and (4) outcomes. LHS frameworks in practice are useful guides for establishing the necessary organizational infrastructure to support continuous improvement and learning.^21^, ^22^

Despite the complexity of different health organizations' governance, funding, and local setting, LHS frameworks are applicable across diverse settings, informing intervention design and strategies instigating organizational change. One academic health center demonstrates the practicality of a LHS framework guiding the design of organizational‐level interventions across five domains including: goals and strategies, culture, people and processes, learning infrastructure, and technology.^23^ Findings show the value of a LHS framework in coordinating structures and processes to achieve organizational improvement, providing insight about the infrastructure required to support organizational competencies for continuous learning.^23^ Applying a LHS framework to implement organizational change, including structures and processes tailored to local settings and organizational priorities, show promise for advancing climate‐resilient and low‐carbon organizational change. Further, principles of environmental sustainability—that is, considering environmental, economic, and social concerns in meeting the needs of the present without compromising the ability of future generations to meet their own needs^24^—provide opportunities for strengthening LHS outcomes including improving high‐quality care, patient and provider experiences, and reducing health care costs. Multiple social, economic, and ecological co‐benefits can be gained from high‐performing, climate‐resilient LHS. For example, identifying resource‐ and emission‐intensive priority areas from the delivery of services can inform interventions to improve efficiency, cost‐effectiveness, and better patient outcomes. Health centers in Spain,^25^ Finland,^26^, ^27^ and Sweden^28^ demonstrate co‐benefits of eliminating duplicate operative services through the implementation of immediate bilateral cataract surgery—phacoemulsification with intraocular lens placement in both eyes of the patient on the same visit in the same operative room—resulting in reduced costs, reduced GHG emissions from less patient travel and building energy use, and faster patient rehabilitation.^29^ Despite common goals of LHS continuous learning and improvement and co‐benefits of high‐performing climate‐resilient health systems, principles of environmental sustainability have not yet been integrated within LHS frameworks.

Further, LHS frameworks provide opportunities to address organizational barriers to climate‐resilient and low‐carbon healthcare by tailoring interventions within LHS domains.^19^ A scoping review of barriers and enablers to implementing environmentally sustainable health systems identifies key organizational barriers, including: individual lack of knowledge and skills; institutional lack of defined targets, investment, and leadership; geographical/infrastructural aging buildings and societal values of environmental stewardship; and system‐level lack of incentives and sustainable principles embedded within clinical guidelines and decision‐making practice.^30^ Key levers supporting coordinated action include the use of data to identify significant sustainability issues, monitoring of GHG emissions at the organizational level, and embedding climate‐resilient and low‐carbon decision‐making in practice.^30^ Identified barriers and levers are similar to characteristics of LHS framework domains, such as Lavis et al.^19^ characteristics of a LHS framework. Further research is required to explore how LHS frameworks could be used to address health system challenges, strengthening planning and coordination of environmentally sustainable LHS.

We aim to address this knowledge gap by describing experiences of planning and coordinating an environmentally sustainable LHS in Nova Scotia, Canada. This experience report describes health system leaders' and healthcare professionals' perspectives on coordinating organizational structures and processes for climate‐resilient and low‐carbon health care and recommendations for advancing an environmentally sustainable LHS framework. The overarching research question guiding this work is how can LHS frameworks strengthen environmentally sustainable organizational structures and processes? To answer this research question, we aim to address the following:

1. What are organizational level challenges to advancing environmentally sustainable LHS?
2. What strategies do key informants recommend for planning environmentally sustainable LHS?

## 2 METHODS

Environmental scan methods^31^ are used to better understand key informants' experiences^32^ for advancing environmentally sustainable health systems. Environmental scans are useful for identifying and assessing information from internal and external environments of an organization, informing strategic decision‐making, and future organizational action.^33^ Environmental scans are particularly relevant for health systems as a tool enabling needs‐assessment, program development, and organizational strategies for implementing change.^34^, ^35^ There is no one established methodology for conducting an environmental scan.^31^ The most common data collection methods for environmental scans include literature reviews, key informant interviews, and surveys.^31^

Nova Scotia, a province in eastern Canada, has a population of approximately 1 million people.^36^ The population is primarily concentrated in urban areas, a majority residing in the Halifax Regional Municipality, the province's largest city.^36^ The remainder of the population is spread across rural communities, which make up a significant portion of the province's land area. Nova Scotia's population is diverse, with an increasing proportion of immigrants, particularly from countries like the Philippines, India, and China.^36^ Immigration plays a crucial role in the province's population growth and demographic shifts. The province's healthcare system is publicly funded and provincially mandated by the Nova Scotia Health Authority's regional health zones: Western, Northern, Eastern, and Central.^37^ This system ensures that residents have access to essential health services, although rural areas may face challenges related to access and availability of care. There are two tertiary care centers in Halifax Regional Municipality, delivering advanced and highly specialized care for complex patients. This study was conducted at one tertiary care center specializing in delivering care to women, children, youth and families in the Maritime provinces. This project was exempt from requiring research ethics approval by the Institutional Research Ethics Review Board to conduct informal discussions as part of a quality improvement project. This project was determined to fall within the category of routine operational improvement and negligible risk or impact involving existing collections of non‐identifiable data.

Key informants were recruited between February and May 2023 during virtual clinical managers council meetings and grand rounds. Five presentations were made by the first author (BB) describing the impact health systems have on climate change and providing examples of evidence‐based low‐carbon health care interventions to mitigate direct and indirect emissions. Following each presentation, all healthcare professional attendees (i.e., executive leaders, clinical managers, medical students, and specialists from anesthesiology, neurosurgery, gastroenterology, neonatal, family medicine, operative services, respirology, emergency, laboratory geneticists, and pharmacy) were invited to discuss their experiences of coordinating health system structures and processes to improve low‐carbon health care and recommendations for advancing environmentally sustainable LHS. There were no inclusion or exclusion criteria limiting participation in group or one‐on‐one discussions. The first author, BB facilitated group discussions following a semi‐structured interview guide, developed using five domains of a LHS framework.^19^ Questions addressed key informants' perceptions of environmental sustainability, knowledge and/or participation in past and current environmental sustainability projects, patient and family engagement in sustainability initiatives, use of data to inform decision‐making, strategic priorities, challenges of achieving environmentally sustainable organizational change, and recommendations for advancing environmentally sustainable organizational strategies. Detailed notes were taken during discussions, and attendees were invited to contact BB to discuss further in a one‐on‐one discussion. All informal group and one‐on‐one discussions were conducted virtually via Microsoft Teams meeting. The first author (BB) conducting discussions has expertise in health systems change and environmentally sustainable healthcare and supported by a research team with expertise in LHS frameworks (CC and JC), and clinical and executive health system leadership (DS).

### 2.1 Data analysis

#### 2.1.1 Guiding framework

This work was guided by Lavis et al.^19^ rapid LHS framework outlining four domains and seven characteristics of a LHS including: (1) engaged patients; (2) digital capture, linkage and timely sharing of relevant data; (3) timely production of research evidence; (4) appropriate decision supports; (5) aligned governance, financial and delivery arrangements; (6) culture of rapid learning and improvement; and (7) competencies for rapid learning and improvement.

#### 2.1.2 Content analysis

Detailed notes were transcribed from informal discussions and analyzed using deductive content analysis.^38^ Transcripts were reviewed by BB and statements were categorized into seven characteristics of a LHS. Categorized statements were reviewed by another researcher on the team, CC, and interpreted by both BB and CC to generate themes.^39^ Emerging themes were synthesized into key challenges and recommendations for advancing environmentally sustainable health systems and mapped onto seven characteristics of a LHS.^19^ Draft results were circulated to clinical managers councils and grand rounds, inviting all attendees to review emergent themes to ensure accuracy and validity and the opportunity for refinement of results based on their feedback.

## 3 RESULTS

Informal discussions, lasting approximately 45 min, were held with 16 health system leaders and healthcare professionals at one tertiary care center. Key informants discussed seven key challenges and recommendations for advancing environmentally sustainable LHS (Table 1) including: (1) patient involvement in co‐creating environmentally sustainable organizational goals and strategies; (2) interprofessional collaborations and organizational partnerships to improve timely sharing of best available evidence, resources, and tools for measuring sustainability metrics; (3) embedded project teams to support timely production of emissions data and implementation of low‐carbon health care; (4) centralized data management system informing low‐carbon decision‐making; (5) embedded research scientists to support identification of evidence‐based low‐carbon practice and enhance a culture of rapid learning and improvement; (6) environmental sustainability values are stewarded by health system leaders and healthcare professionals at all levels; (7) organizational training and resources to improve knowledge and competencies of climate‐resilient and low‐carbon health systems.

**TABLE 1:** Challenges and recommendations for advancing environmentally sustainable learning health systems.

LHS category^19^	LHS characteristics^19^	Challenges	Recommendations
Patient centered	**Engaged patients:** Systems are anchored on patient needs, perspectives, and aspirations (at all levels) and focused on improving their care experiences and health at manageable per capita costs and with positive provider experiences	Patients and communities are currently not engaged in identifying organizational environmental sustainability priorities. A planetary health framework is not currently integrated within patient‐centered approaches to health care.	Patients and communities are valuable partners for co‐creating a shared vision of environmentally sustainable health care and identifying opportunities to improve climate‐resilient and low‐carbon health systems. Patients and communities should be informed of environmental impacts from delivery of care and inappropriate use of health care services contributing to emissions (i.e., unnecessary testing and treatment). It is recommended patients and communities are engaged in co‐creating environmentally sustainable organizational goals and strategies for mitigating environmental impacts during health care interactions, such as opportunities for reducing unnecessary hospital visits using virtual care and coordinating health care closer to home in community clinics that reduce demand for emission intensive ambulatory care and hospital‐based treatments.
Data and evidence driven	**Digital capture, linkage and timely sharing of relevant data:** Systems capture, link and share (with individuals at all levels) data (from real‐life, no ideal conditions) about patient experiences (with services, transitions and longitudinally) and provider engagement alongside data about other process indicators (e.g., clinical encounters and costs) and outcome indicators (e.g., health status)	Environmental impacts from direct and indirect sources of emissions are not systematically measured and monitored across health organizations and health authorities.	Sustainability data on direct and indirect sources of emissions should be routinely collected and reported as part of organizational goals and risk assessments to address sustainability challenges. It is recommended interprofessional collaborations and partnerships with other organizations are leveraged to improve timely sharing of best practice, lessons learned, and approaches for measuring sustainability metrics. The Canadian Coalition for Green Healthcare^40^ and Center for Sustainable Health Systems (CASCADES)^41^ provide essential resources and toolkits for health system leaders and healthcare professionals. The Coalition's Green Hospital Report Card toolkit offers a standardized approach for assessing, measuring, and reporting institutional emissions on energy and water conservation, waste management and recycling, corporate commitment, and pollution prevention. Organizations can compare year‐over‐year environmental performance against other participating health organizations.
	**Timely production of research evidence:** Systems produce, synthesize, curate and share (with individuals at all levels) research about problems, improvement options and implementation considerations	Low‐carbon health care practice changes are often siloed to clinical units and challenged with disseminating information and leading sustainable practice across health organizations.	An organizational project team is required to support timely capture and sharing of emissions data to identify high impact areas for low‐carbon adaptations that can be scaled across health organizations. It is recommended an environmentally sustainable project team is embedded within quality improvement, procurement, infection prevention and control, and clinical practice committees to support timely production of emissions data, rapid‐cycle improvement, and well‐coordinated implementation of low‐carbon health care.
System supported	**Appropriate decision supports:** Systems support informed decision‐making at all levels with appropriate data, evidence, and decision‐making frameworks	Organizations are challenged with supporting low‐carbon decision‐making at all levels. Challenges persist in using data to inform environmentally sustainable policies, procedures, and decision‐making in practice, particularly during procurement and at point of care.	A centralized data management system is required to support the use of real time data informing low‐carbon decision‐making in practice. It is recommended a centralized data management system is integrated within existing clinical and administrative workflow to inform emissions associated with healthcare delivery. Nova Scotia is currently implementing a provincial integrated electronic medical record system, providing an opportunity to gather and monitor data on emissions from healthcare delivery (e.g., prescribing, unnecessary diagnostic tests, driving to healthcare appointments) and to support low‐carbon decision‐making for clinicians and health system leaders.
	**Aligned governance, financial and delivery arrangements:** Systems adjust who can make what decisions (e.g., about joint learning priorities), how money flows and how the systems are organized and aligned to support rapid learning and improvement at all levels	Policies and procedures influencing day‐to‐day organizational and clinical practice are not guided by environmentally sustainable frameworks and principles.	An organizational environmental sustainability steering committee and sub‐committees are required to mobilize action such as reviewing and updating policies, procedures, and standards of care that improve low‐carbon practice. It is recommended health organizations dedicate resources to reviewing and revising policies and procedures for improving low‐carbon health care such as within facilities management, pharmacy, procurement, and infection prevention and control teams. Embedded researchers with expertise in knowledge translation and implementation science are well suited to support uptake of evidence‐based low‐carbon interventions and enhance a culture of rapid learning and improvement.
Culture and competencies enabled	**Culture of rapid learning and improvement:** Systems are stewarded at all levels by leaders committed to a culture of teamwork, collaboration, and adaptability	Challenges persist in stewarding sustainability culture and strategic priorities, including limited structures and processes in place supporting rapid learning and improvement, and mobilizing environmentally sustainable organizational change.	Organizational executive leadership is required to champion and steward values of sustainable health systems, advancing a culture of environmentally sustainable rapid learning and improvement. It is recommended a culture of sustainable health systems be advocated for at all levels through widespread training, communication of organizational priorities and goals, reporting of sustainability metrics, and incentives for interprofessional collaboration to achieve sustainability co‐benefits.
	**Competencies for rapid learning and improvement:** Systems are rapidly improved by teams at all levels who have the competencies needed to identify and characterize problems, design data‐ and evidence‐informed approaches (and learn from other comparable programs, organizations, regions, and sub‐regional communities about proven approaches), implement these approaches, monitor their implementation, evaluate their impact, make further adjustments as needed, sustain proven approaches locally, and support their spread widely	Health system leaders and healthcare professionals have limited knowledge of direct and indirect emissions, making it challenging to identify high impact areas and implement evidence‐informed mitigation strategies. Limited awareness of sustainability co‐benefits gained from mitigating environmental harms results in missed opportunities to improve patient care, reduce health care costs, and strengthen climate‐resilient health systems.	A well‐coordinated strategy for increasing organizational knowledge of high‐value and environmentally sustainable healthcare, including increasing internal capabilities for implementing and evaluating evidence‐based practice change. It is recommended organizations develop a multi‐pronged strategy to increase organizational capabilities and knowledge of GHG emissions and low‐carbon alternatives. Strategies for increasing awareness and knowledge include regular town hall education sessions, monthly departmental presentations, online training resources for clinical specialties and staff, and organizational communications strategy to disseminate information and learning opportunities. Increasing internal capabilities for implementing environmentally sustainable practice requires embedded research scientists with expertise in environmental sustainability and implementation science to identify high‐priority areas, barriers and enablers to practice change, evidence‐informed approaches, and implementation and evaluation of low‐carbon practice changes.

Abbreviation: LHS, learning health systems.

## 4 DISCUSSION

There are multiple complex and organizational‐level challenges to supporting the coordination and implementation of climate‐resilient and low‐carbon health care. LHS frameworks^19^ provide structure for identifying and addressing organizational challenges and establishing critical structures and processes to adapt to routinely collected data and lessons learned through rapid cycle improvements.^16^, ^42^ Without integrated structures and processes for leveraging emissions data, such as prescribing or procuring low‐carbon alternatives, health systems will continue to be challenged with developing their own tailored approach for successful uptake of climate‐resilient and low‐carbon health care. Discussions with health system leaders and healthcare professionals provide important insight into the challenges to coordinating low‐carbon health care that is patient‐centered, data and evidence‐driven, system‐supported, and culture and competencies‐enabled.^19^ Recommendations from health system leaders and healthcare professionals highlight opportunities for strengthening environmentally sustainable LHS, reinforcing the importance of enhancing value‐based healthcare (VBHC)—delivering value for patients, relative to costs^43^—through conservation of social, economic, and ecological resources to meet population health needs without compromising the needs of future generations.^24^ Co‐benefits of environmentally sustainable LHS contribute towards achieving the goals of VBHC.

Challenges and recommendations for advancing environmentally sustainable LHS align with findings from a scoping review characterizing barriers and enablers to implementing sustainable health systems^30^ and a systematic review synthesizing the types of implementation strategies to enhance LHS functioning.^44^ Key levers for implementing sustainable health systems include the use of data to identify sustainability issues and priority areas, monitoring of GHG emissions at the organizational level, and embedding low‐carbon alternatives as the default option.^30^ Further, LHS characteristics and implementation strategies highlight the importance of digital infrastructure and changing record systems to implement timely production of data and evidence to enhance LHS functioning.^44^ Discussions with health system leaders and healthcare professionals contribute similar findings, highlighting the importance of a centralized data management system, buy‐in from health system leaders and healthcare professionals at all levels, and embedded environmental sustainability frameworks guiding coordinated decision‐making across administrative and clinical practice areas.

### 4.1 Recommendations for advancing an environmentally sustainable LHS

A central aim of LHS systems is to enhance VBHC by aligning delivery of care to improve outcomes that matter most to patients, relative to the cost of delivering care.^43^, ^45^ Value is created through cycles of continuous improvement driving health system progress, such as integrated health services designed to reduce costs and improve outcomes through comprehensive health services.^43^, ^46^ VBHC evaluations emphasize maximizing the value of each health care transaction; however, this approach does not adequately weigh competing priorities of short‐term financial costs versus the long‐term sustainability of health systems. We recommend that “value” in health care is redefined to include a more comprehensive understanding of costs, including value gained from health system investments in health promotion, disease prevention, and planetary health.

Principles of environmental sustainability can improve our understanding of how to operationalize VBHC, aligned with the “triple bottom line” of health care to improve patient outcomes, reduce health care costs, and mitigate environmental impacts from delivery of care.^47^, ^48^ Furthermore, principles of environmental sustainability have not yet been integrated within the quintuple aim of healthcare, for achieving better population health, improved patient and provider experience, health equity, and reduced health system costs.^49^ It is recommended environmental sustainability is regarded as central to achieving the quintuple aim, strengthening many of the social, economic, and ecological co‐benefits from high‐performing, climate‐resilient, and low‐carbon health systems.^50^

A crucial part of operationalizing VBHC and integrating environmental sustainability principles within LHS frameworks is the use of data and timely production of evidence to measure patient outcomes, quality of care, cost savings, and emissions from delivery of care.^51^ Further research is required to improve linking data between clinical and environmental indicators, such as providing emissions data within electronic medical records to monitor and evaluate the complete financial, environmental, and social costs of every health care interaction. It is recommended digital tools are leveraged to provide real‐time sustainability data, setting in motion organizational structures and processes for making climate‐resilient and low‐carbon alternatives the easy and default decision.

Patient and community engagement are crucial for improving healthcare outcomes and informing environmentally sustainable health care, such as drawing upon patient experiences of climate‐related health conditions and risks.^52^ However, strategies for engaging patients and communities within environmentally sustainable health systems research, such as the co‐creation of climate‐resilient and low‐carbon organizational goals is largely unexplored.^44^ It is recommended patients and communities are engaged as equal partners in improving environmentally sustainable healthcare, especially for mitigating environmental impacts that disproportionately affect marginalized communities at increased risk of climate‐related diseases, injuries, and displacement.^53^ Furthermore, investigating unmet patient needs could identify what community‐based interventions would help to mitigate environmental impacts such as improving access to more appropriate community‐based resources that reduce demand for emission‐intensive health services from ambulatory care and hospital‐based treatments.

In‐depth insights about challenges and recommendations for coordinating and planning environmentally sustainable LHS provides an important starting point for understanding how principles of environmental sustainability can advance LHS frameworks. However, generalizability of results is limited due to a small sample size of health system leaders and healthcare professionals at one tertiary care center. Key informants' discussion of challenges and recommendations is relevant to the context of Nova Scotia, a province in the early stages of developing climate change adaptation strategies. Results may not be generalizable to health system settings that are further along in developing climate change adaptation strategies.

Results of this study are structured based on characteristics of a LHS framework^19^ and limited from LHS focus on values versus outcomes, short‐term versus long‐term priorities, requisite of organizational readiness for change, and internal capabilities to maintain health system change. Key informants' recommendations are limited in focus on long‐term health system change, requiring time and resources to address multi‐level challenges, especially for health organizations with fewer resources guiding *how* to coordinate organizational change and limited internal capabilities to implement evidence‐based environmentally sustainable practice.

Additional research is required to investigate how LHS frameworks can support environmentally sustainable health transformation, such as identifying the right mix of team members, leaders, and embedded research scientists across administrative and clinical teams, integration of environmental sustainability principles informing decision‐making in practice, and effective implementation strategies for advancing environmentally sustainable organizational culture, knowledge, and competencies. Despite evidence of sustainability co‐benefits for improving patient care and reducing emissions from care,^54^ transforming environmentally sustainable health care involves large‐scale effort to successfully implement organizational changes.^51^ Health organizations at the early stages of improving environmentally sustainable structures and processes would benefit from using an evidence‐based framework, such as LHS, to assess organizational readiness for change.^55^ LHS frameworks can help speed up environmentally sustainable organizational change by identifying priority areas of action and supporting the uptake of evidence‐based climate‐resilient and low‐carbon alternatives.

## 5 CONCLUSION

LHS frameworks provide a structured approach for coordinating organizational change and identification of strategies for implementing environmentally sustainable practices. LHS frameworks provide a practical guide for coordinating organizational change; however, LHS frameworks can be strengthened by principles of environmental sustainability that emphasize VBHC outcomes and long‐term priorities for health system change. Principles of environmental sustainability provide insight for strengthening LHS functioning and the long‐term economic and ecological viability of health systems. This environmental scan highlights seven challenges and recommendations for advancing environmentally sustainable LHS. Further research is required to build the evidence base with practical guidance on how to implement environmentally sustainable LHS.

## FUNDING INFORMATION

This study was funded by the Canadian Institutes of Health Research, Health Systems Impact Postdoctoral Fellowship Award (202203IF9‐483714‐294387).

## CONFLICT OF INTEREST STATEMENT

All authors declare that they have no conflicts of interest.
